# Critical evaluation of food intake and energy balance in young modern pentathlon athletes: a cross-sectional study

**DOI:** 10.1186/s12970-016-0127-x

**Published:** 2016-04-02

**Authors:** Leticia Azen Alves Coutinho, Cristiana Pedrosa Melo Porto, Anna Paola Trindade Rocha Pierucci

**Affiliations:** Federal University of Rio de Janeiro/Josué de Castro Nutrition Institute, Av. Carlos Chagas Filho, 373 - Centro de Ciências da Saúde, Bloco J, 2° andar, Cidade Universitária, Ilha do Fundão, RJ 21941-902 Brazil

**Keywords:** Adolescents, Sports nutrition, Physical exercise, Body composition, Eating habits

## Abstract

**Background:**

Modern pentathlon comprises five sports: fencing, swimming, equestrian jumping, and a combined event of pistol shooting and running. Despite the expected high energy demand of this sport, there are few studies that provide support for the nutritional recommendations for pentathletes. The purpose of the present study was to evaluate young modern pentathlon athletes with respect to body composition, biochemical profile, and consumption of food and supplements.

**Methods:**

Fifty-six young modern pentathletes aged 13.5 ± 2.4 years participated in the study: 22 adolescent girls and 34 adolescent boys, weight 55.8 ± 13.3 kg, height 1.6 ± 0.1 m, and body fat 21.1 ± 3.1 %. Food consumption was analyzed through a 24-h recall method and food-frequency questionnaire. Assessment of body composition was carried out by checking anthropometric measures (body mass, height, and skinfolds) and using protocols according to participants’ age and sexual maturity.

**Results:**

Male participants consumed less energy than the general recommendations for athletes from the American Dietetic Association (2749 ± 1024 kcal vs. 3113 ± 704 kcal, *p* < 0.01), whereas female participants consumed more energy than those recommendations (2558 ± 808 kcal vs. 2213 ± 4734 kcal, *p* < 0.01). Neither young men nor young women followed the carbohydrate intake recommendations for athletes (6.3 ± 2.5 g/kg/day and 6.6 ± 2.2 g/kg/day, respectively). Lipid and protein intakes corresponded to recommendations for both sexes; however, insufficient intakes of calcium, fruits, and vegetables were seen, as well as frequent consumption of baked goods and sugared soft drinks.

**Conclusions:**

Adolescent modern pentathlon athletes presented inadequate eating habits with respect to consumption of carbohydrates and energy. Many participants had insufficient intake of micronutrients, especially calcium. However, future research is needed that is aimed at elucidating the real nutritional demands for good physical performance in this sport and the impact of inadequate eating habits on performance, especially among young athletes who are in the growth-stage years and are exposed to intense physical exercise routines.

## Background

The modern pentathlon is an Olympic sport that involves five modalities [1]. Modern pentathlon competitions last for about 8 h and include the sequential practice of sword fencing (all against all), 200-m freestyle swimming, equestrian show jumping (horses randomly chosen for competition) and finally, a combined event of pistol shooting (five well-aimed shots at the target) and running (four 800-m run cycle) [1].

In Brazil, the modern pentathlon is still not well known but has a significant role in social projects promoted by the Brazilian Modern Pentathlon Confederation (acronym in Portuguese: CBPM), with a predominance of adolescents among its participants.

The practice of competitive sports by adolescents requires special attention owing to their biological stage in which significant body changes related to sexual maturation and growth take place [2, 3]. Adolescent athletes’ nutrition must promote adequate growth and development in addition to meeting the increased nutritional demands of strenuous physical activity. However, studies have shown that the intake of certain quantities of energy and nutrients by young athletes is below recommendations [2, 3].

In general, adolescent athletes often show insufficient intake of calcium, thereby becoming susceptible to low bone density and stress fractures [4]. It has been suggested that track and field athletes consume high amounts of lipids, saturated fats, and mono- and disaccharides whereas their iron intake is usually lower than recommendations, especially in women [5]. Such deficient energy balance and insufficient nutrient intake may impair the growth, health, and physical performance of these athletes. Thus, young athletes constitute a population that is vulnerable to the physiological effects of chronic physical fatigue owing to intense exercise, especially if they have inadequate food consumption.

Recently, Le Meuer et al. [6, 7] evaluated physiological demands during the new combined event (sport shooting and running) among adult modern pentathletes. Their most important finding was that the athletes performed this event close to their VO_2_ max. However, there is currently little scientific literature [1] in this area; therefore the metabolic demands of other pentathlon events have not been established.

In the shooting and equestrian portions of the pentathlon, a static form of strength performance with low energy demands can be identified whereas events of a cyclic nature, such as running and swimming, have characteristically high demands on athletes’ energy systems [8]. Fencing is a combat sport that is characterized by open skills and a noncyclic type of intermittent load, which requires high levels of agility and athlete concentration [9].

Because neither the metabolic requirements nor nutritional practices of the modern pentathlon have been established, and the required anthropometric profile for this sport is well known, the main purpose of the present study was to critically evaluate food consumption among young pentathletes, considering the general recommendations for athletes of the American Dietetic Association (ADA) [10]. Our hypothesis was that young pentathletes present nutritional inadequacies, especially with respect to intakes of energy, lipids, and micronutrients, and that young men and women have different eating habits, as observed in other studies involving adolescents.

## Methods

### Subjects

Fifty-six healthy young athletes voluntarily took part in this study. There were 22 adolescent females and 34 adolescent males, all affiliated with the Modern Pentathlon Federation of the State of Rio de Janeiro (FPMERJ, acronym in Portuguese). Participants were chosen by their coach as being diligent in training with the “PentaJovem” (acronym in Portuguese) team. The inclusion criteria were as follows: a) having trained for at least 6 months; and b) aged between 10 and 18 years (classified as postpubescent, according to sexual maturity). All adults were excluded.

The research was approved by the Committee of Ethics in Research of Clementino Fraga Filho University Hospital of the Rio de Janeiro Federal University (Protocol No. 90/11) and was carried out as per the norms in Resolution No. 196/96 of the National Health Council, which issues directives and regulations on the use of human beings in research.

### Antropometry and sexual maturity

One experienced researcher carried out the anthropometric evaluations. Body mass and height were measured in accordance with the criteria of Gordon et al. [11], using a medical scale with a stadiometer (Welmy™, Brazil) ranging from 0 to 150 kg, with precision 100 g.

Two cutaneous skinfolds (triceps and subscapular) were measured in triplicate on the right side of the body, according to Harrison et al. [12], using an adipometer with 10 g/mm^2^ constant pressure (Lange™, USA) and precision 1 mm. A new set of measurements were taken if there was >5 % disagreement in one of the three measurements. The final result was expressed as an arithmetic mean of the three measurements.

Evaluation of athletes’ sexual maturity was carried out using the criteria proposed by Tanner, which divides adolescence into five phases, starting at the prepubertal stage (stage 1), going through puberty (stages 2 to 4), and finishing at the postpubertal stage (stage 5). Evaluation was made by showing participants photos that referred to the adolescent developmental stages of pubic hair growth for both sexes, breast development for adolescent girls, and genital development adolescent boys [13].

Body mass index was calculated with AnthroPlus software of the World Health Organization, and results were compared with the reference distribution [14]. Body density and body fat percentage were estimated using the equation of Slaughter et al. [15].

### Biochemical profile

After participants had completed a 12-h fast, a qualified nurse drew blood samples from the antecubital vein. Participants had been instructed not to engage in any physical activity for the 24 h prior to sample collection and to abstain from consuming alcoholic beverages for the previous 72 h. Blood counts and blood lipid profiles were automatically analyzed (Lab Max Plenno Labtest™), as well as glucose (Citometro Cell-Dyn 1700 Abbott™), using Diagnostica™ Labtest kits.

### Energy expenditure

To estimate the total energy expenditure (TEE), methods proposed by Iglesias-Gutierrez et al. [16] and Leenders et al. [17] were adapted, considering three components: 1) basal metabolic rate (BMR), calculated using the FAO/WHO/UNU equation [18]; 2) thermic effect of food (10 % of TEE); and 3) energy expenditure (EE) related to routine activities and physical training.

To calculate routine activity EE, athletes or their parents were asked to register participants’ daily activities based on the questionnaire proposed by Bouchard et al. [19]. Team trainers recorded data of physical training (duration, distances covered, and intensity of each activity, as per the Rated Perceived Exertion Scale [20]. Metabolic equivalents [21] were used to quantify EE for each physical activity.

### Food and supplements consumption

Athletes' food consumption was evaluated by a 24-h recall method (R24h) and food-frequency questionnaire (FFQ) for adolescents. Athletes’ use of supplements was registered on a separate form, specifying the type, purpose, manner of use, and origin of the indication.

Quantitative analysis of ingested energy and nutrients (carbohydrates, proteins, lipids, calcium, iron, and vitamins A and C) for each meal reported by participants in the R24h was carried out, by assessing nutritional composition in accordance with the Table of Food Composition of the Brazilian Institute of Geography and Statistics [22] and the nutritional information on food labels for foods not listed in the reference table.

Assessment of the adequacy of macronutrient intake was made based on the publication “Nutrition and Athletic Performance” of the ADA [10]. Dietary reference intake (DRI) [23] values were considered to assess the adequacy of micronutrient intake. Intake was considered inadequate for values less than the estimated average requirement (EAR) or greater than the Tolerable Upper Intake Level.

To interpret results of the FFQ, foods were grouped on the basis of food groups set as healthy food markers in the Consumer Expenditure Survey 2008–2009 [24]. Frequency of consumption categories considered were: once or less per week, two to four times per week, and five times per week or more.

### Statistical analyses

The results are expressed in averages (±standard deviation). The normality of the data was checked using the Shapiro–Wilk test. To compare independent data, the t-test was chosen for independent samples, at statistical significance level *p* < 0.05. All analyses were performed using IBM SPSS software™ version 20.0 (Armonk, NY, USA).

## Results

Results related to anthropometry and sexual maturity are shown in Table 1. The findings showed that 78.5 % of participants, for both sexes, were in the pubertal developmental stage. Adolescent girls showed a higher body fat percentage in the pubertal and postpubertal stages than their male counterparts.

**Table 1:** Anthropometric profile of modern pentathlon athletes (*n* = 56), according to the stages of sexual maturation and gender, X ± SD

	Male (*n* = 34)			Female (*n* = 22)		
	Prepubescent	Pubescent	Post pubescent	Prepubescent	Pubescent	Post pubescent
	(*n* = 1)	(*n* = 27)	(*n* = 6)	(*n* = 2)	(*n* = 17)	(*n* = 3)
Weight (kg)	39.4	58.49 ± 12.5	70.8 ± 9.9	34.6 ± 6.9	49.2 ± 9.9	64.2 ± 6.3
Height (cm)	148.0	163.4 ± 10.3	175.9 ± 7.4	146.0 ± 5.6	155.5 ± 10.7	162.6 ± 3.5
BMI (kg/m^2^)	18.0	21.68 ± 3.2	22.8 ± 1.9	16.1 ± 1.9	20.1 ± 2.6	24.3 ± 2.4
Body fat (%)	20.0	20.4 ± 8.3	15.5 ± 6.4	16.35 ± 0.7	23.8 ± 6.8	28.4 ± 1.2

*BMI* body mass index

The assessed laboratory parameters were generally within normal ranges for all athletes in this study. The following average results were observed: hemoglobin 13.5 ± 1.2 g/dl; hematocrit 42.0 ± 3.4 %; glucose 90.4 ± 6.5 mg/dl; total lipids 421.7 ± 71.6 mg/dl; total cholesterol 151.9 ± 25.2 mg/dl; HDL cholesterol 52.4 ± 12.7 mg/dl; LDL cholesterol 83.5 ± 23.1 mg/dl; and triglycerides 79.5 ± 5.1 mg/dl.

**Fig. 1:**
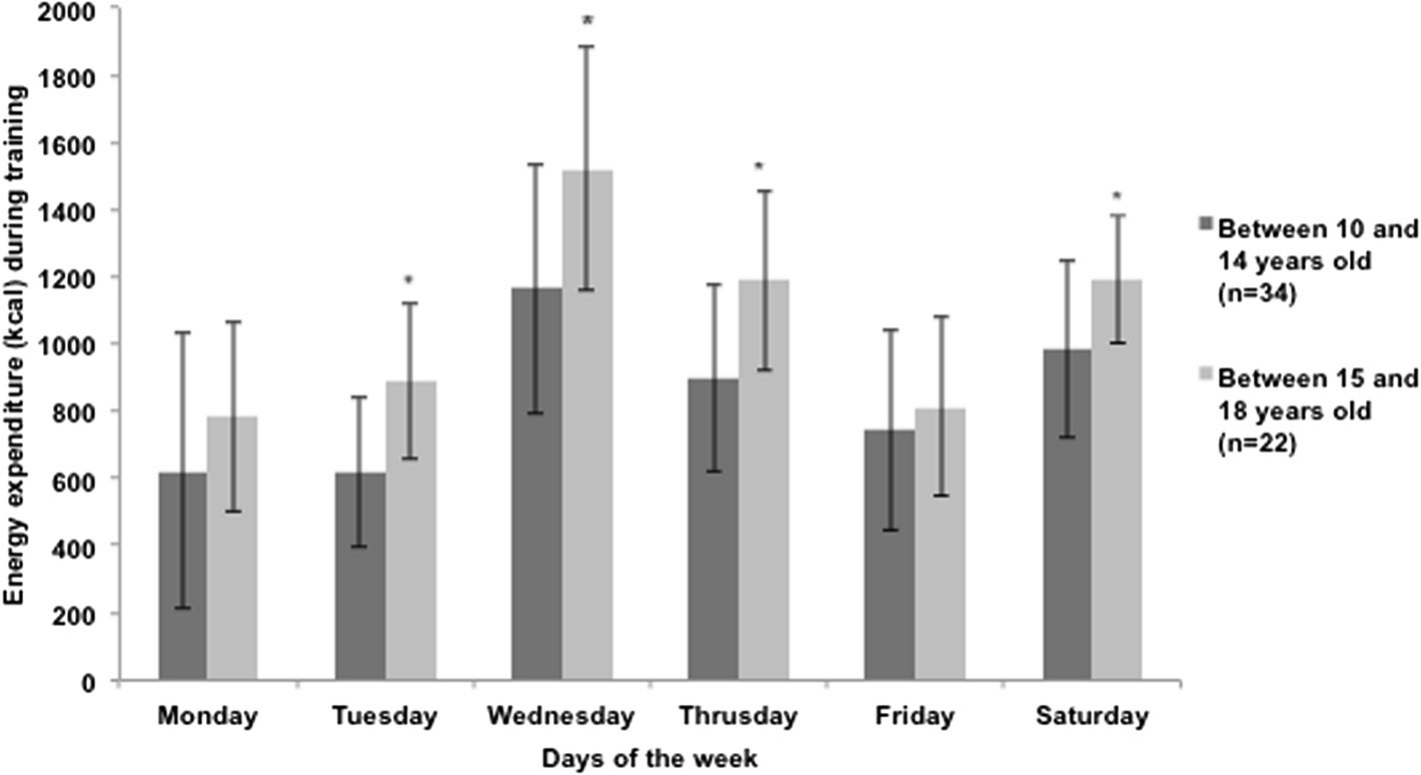
Weekly energy expenditure (X ± SD) during training performed by modern pentathlon athletes, PentaJovem team, differentiated by age. * Significant difference (*p* <0.05) from the athletes older than 15 years old, t-test for independent sample

Values of estimated EE were determined using records of athletes’ physical training routines. The youngest athletes (aged between 10 and 14 years and belonging to the Young E10, Young D, Young C categories) had lower EE values compared with the oldest ones (over 15 years old and belonging to the Young B and Young A categories); these results were based on training three times a week, with older athletes training about 3 h a day and younger ones training about 2 h a day. For athletes in both these age groups, EE tended to increase from Monday through Wednesday, subsequently decreasing through Friday, and increasing again on Saturday (Monday, 619 ± 410 kcal vs. 782 ± 280 kcal; Tuesday, 617 ± 223 kcal vs. 887 ± 231 kcal; Wednesday, 1163 ± 371 kcal vs. 1520 ± 363 kcal; Thursday, 896 ± 275 kcal vs. 1188 ± 268 kcal; Friday, 742 ± 296 kcal vs. 812 ± 264 kcal; Saturday, 982 ± 262 kcal vs. 1193 ± 191 kcal, for younger and older athletes, respectively) (Fig. 1).

With respect to supplement intake, 35 (62 %) athletes reported the use of some type of nutritional supplement, but only 6 % used supplements as prescribed by a nutritionist. The majority (46 %) used supplements as suggested by their trainer, emphasizing the fact that 86 % of athletes had not received any professional nutritional counseling.

Energy supplements, hydroelectrolytic supplements, vitamin C, and multivitamins were the most frequently consumed supplements, corresponding to 31 %, 25 %, 19 % and 15 % of the products reported by participants, respectively.

Although the difference between adolescent boys and girls was not significant, Table 2 shows that male athletes tended to have greater energy intake than their female counterparts. However, boys did not reach the estimated TEE, whereas girls had higher energy intake than recommendations. In general, macronutrient intake did not differ between boys and girls, whereas intakes of proteins and lipids were adequate. However, both male and female athletes consumed fewer carbohydrates (*p* < 0.01) than the average recommendations of the ADA (2009) [10].

**Table 2:** Intake of macronutrients and energy by modern pentathlon athletes (*n* = 56), according to gender, X ± SD

Variables	Female (*n* = 22)		Male (*n* = 34)	
	ADA (2009) recomendations	Intake	ADA (2009) recomendations	Intake
Energy (kcal)	2213 ± 473^a^	2558 ± 808^b^	3113 ± 704^a^	2749 ± 1024^b^
Protein (g/kg)	1.2–1.7	1.7 ± 0.6	1.2–1.7	1.6 ± 0.5
Carbohydrate (g/kg)	6.0–10.0	6.6 ± 2.2^c^	6.0–10.0	6.3 ± 2.5^c^
Lipids (%VET)	25.0–35.0 %	30.3 ± 6.6	25.0–35.0 %	31.4 ± ± 8.4

^a^Estimated values according to Iglesias-Gutierrez et al. (2005) [16] and Leenders et al. [17]

^b^Significant difference compared to the estimated energy expenditure (*p* <0.01)

^c^Significant difference compared to the ADA (2009) [10] recommendations (*p* <0.01), t-test for independent samples

Considering the energy intake for each meal as a percentage of the consumed total energy value, it was found that the athletes concentrated their largest intake of energy in the main meals (breakfast, lunch and dinner) with 20 % consumed at breakfast, 7 % with mid-morning snacks, 27 % at lunch, 5 % during physical activity, 15 % after physical activity, 25 % at dinner, and 1 % at supper.

According to the needs of each sex and age group, we evaluated the adequacy of micronutrient intake (Table 3). In general, both male and female athletes showed a high proportion of inadequate vitamin A and C intake. Nearly all athletes consumed inadequate amounts of calcium; however, most athletes of both sexes and in all age groups consumed adequate quantities of iron.

**Table 3:** Average intake of micronutrients and distribution of pentathletes (%) (*n* =56) on the adequacy of micronutrient intakes, according to gender and age

	Vitamins								Minerals							
	Vitamin A				Vitamin C				Calcium				Iron			
Gender/Age	Intake (mcg)	<EAR	≥EAR ≤ UL	>UL	Intake (mg)	<EAR	≥EAR ≤ UL	>UL	Intake (mg)	<AI	≥AI ≤UL	>UL	Intake (mg)	<EAR	≥EAR ≤ UL	>UL
Male																
10–13 (*n* = 13)	551.5 ± 1207.7	92.3 %	–	7.7 %	100.2 ± 120.6	61.5 %	38.5 %	–	312.3 ± 181.5	100.0 %	–	–	11.6 ± 5.5	23.0 %	77.0 %	–
14–18 (*n* = 21)	870.1 ± 1872.8	80.9 %	14.2 %	4.9 %	76.3 ± 114.8	66.7 %	33.3 %	–	510.5 ± 347.8	95.2 %	4.8 %	–	13.4 ± 5.2	14.3 %	85.7 %	–
Female																
10–13 (*n* = 12)	1026.3 ± 2508.5	75.0 %	16.7 %	8.3 %	29.6 ± 51.3	83.3 %	16.7 %	–	344.1 ± 243.7	100.0 %	–	–	11.7 ± 8.9	8.3 %	91.7 %	–
14–18 (*n* = 10)	1248.8 ± 2777.3	70.0 %	20.0 %	10.0 %	117.1 ± 107.6	50.0 %	50.0 %	–	479.2 ± 271.0	100.0 %	–	–	12.4 ± 4.0	10.0 %	90.0 %	–

*EAR Estimated Energy Requirement*, *UL Tolerable Upper Intake Level*, *AI Adequate Intake*

**Fig. 2:**
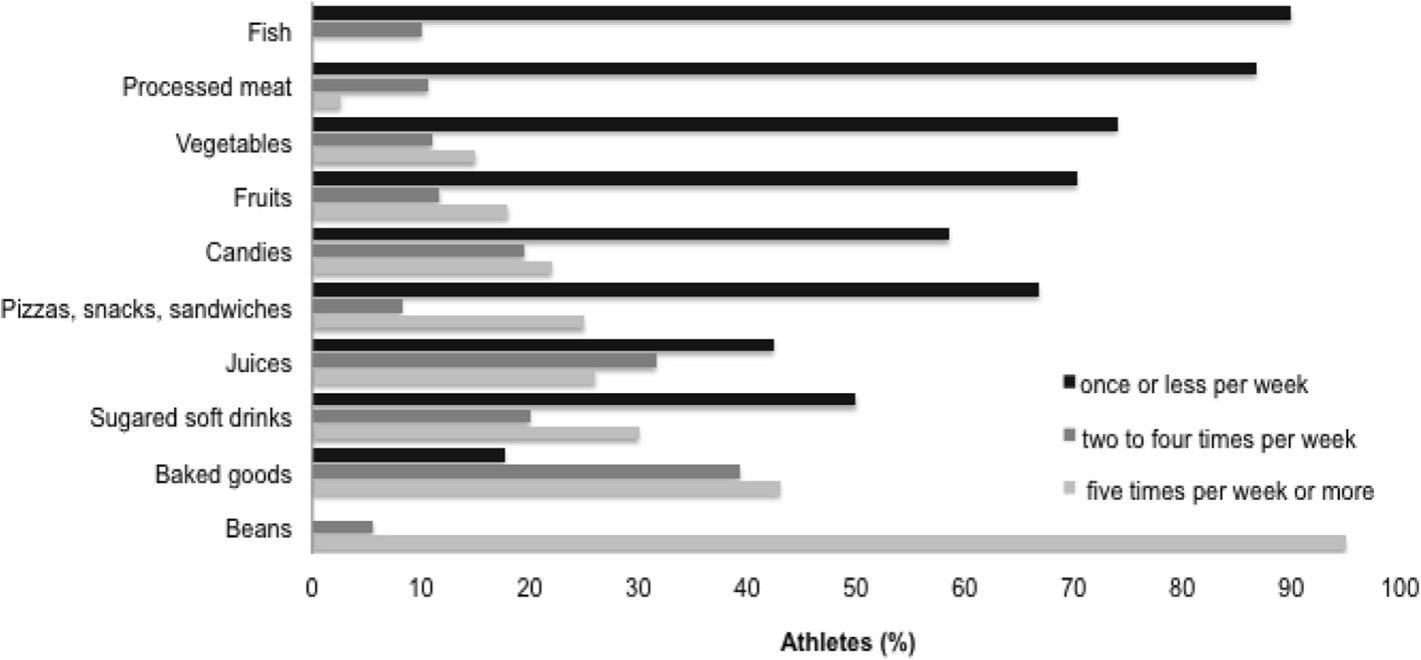
Frequency (%) of athletes who consume each groups of food set as healthy markers according to Consumer Expenditure Survey 2008–2009 [24]

The results of qualitative analysis of participants’ eating habits, evaluated by means of an FFQ, are shown in Fig. 2. Legumes were consumed by 95 % of athletes, baked goods by 43 %, and sugared soft drinks by 30 %, all with a frequency of five times a week or more. On the other hand, 90 % of athletes consumed fish, 87 % ate processed meats, and 74 % consumed vegetables, all with a frequency of once or less per week.

## Discussion

For optimal performance in sports, adequate nutrition and physical training are essential factors. There are few scientific studies on this subject that involve pentathletes, so knowledge remains insufficient regarding the physical demands of these athletes and the ideal nutritional habits necessary to improve physical performance. This is the first study of adolescent modern pentathlon athletes to assess body composition, biochemical profile, and consumption of food and supplements.

Body composition is an important indicator of physical fitness and the general health of athletes [8]. The study Claessens et al. [25] among 54 adult female pentathletes (average body weight 61 ± 5.3 kg, body fat 16 ± 2.4 %) revealed an inverse relationship between fat mass and pentathlon performance.

Considering the incompleteness of body mass index data in terms of determining body composition variability and changes in the proportions of fat mass and fat-free mass, it becomes necessary to analyze the body composition of elite pentathletes in the form of component structures. Generally, lower fat mass proportion, greater musculature, and more active mass are required in most sports disciplines [8].

Our research group previously assessed the body composition of elite pentathletes using dual-energy x-ray absorptiometry and found substantial effects of sports activities on anthropometry results [26], especially among men. On the other hand, the optimal body composition for a specific sport discipline is difficult to determine^8^.

Cech et al. [8] aimed to describe the current profile of body composition among elite young male and female modern pentathletes. They detected sex differences in that men had a higher proportion of fat free mass (women, 52.6 ± 3.5 kg vs. men, 66.4 ± 3.3 kg) and less fat mass (women, 15.8 + 1.38 % vs. men, 8.8 + 0.7 %). According to the authors, their results were in accordance with the published literature. In the present study, we also observed that adolescent girls had a higher percentage of body fat compared with adolescent boys. However, despite finding higher results for both sexes, our findings could not be compared with those of Cech et al. [8] because body fat was predicted in that study using a different technique (bioelectrical impedance analysis).

Nutritional needs during adolescence have a stronger relationship with physiological age than with chronological age and are thus directly proportional to the speed of growth and changes in body composition. Therefore, chronological age alone should not be used as an indicator of adolescent developmental state because individuals of the same age differ in their stages of sexual maturation [27, 28]. In the present study, we confirmed the importance of evaluating the stage of sexual maturity together with an evaluation of body composition, particularly in adolescent girls between 13 and 15 years old who are classified as either pubertal or postpubertal.

Adolescents require special attention during this biological period, which includes noticeable body changes related to sexual maturity and growth. However, it is known that the eating habits of adolescents are frequently inadequate. Adolescents often substitute meals with snacks of low nutritional value [29] and consume insufficient amounts of milk, dairy products, fruits, and vegetables [30–32], as well as large amounts of high energy-density foods that are rich in sodium and sugar, such as soft drinks and fast foods [31, 33, 34].

Our qualitative analysis of participants’ eating habits showed that the most frequently consumed foods (five times a week or more) were legumes, baked goods, and sugared soft drinks. Few pentathletes ate vegetables and fruits with this same frequency. Our findings therefore corroborate previous findings demonstrating that young athletes have inadequate nutritional intake levels, particularly with respect to energy, carbohydrates, vitamins A and C, and calcium. However, intakes of lipids, proteins, and iron among this population were adequate.

In the present study, a deficit in total energy intake among adolescent males was verified, different to the findings of Braggion et al. [35], who observed energy intake below DRI values among both male and female adolescent athletes. Moreover, Kazapi and Ramos [30] observed a greater prevalence of restricted energy intake among female athletes than their male counterparts. According to Panza et al. [36], many athletes who engage in different sports, especially female athletes, usually adopt dietary restrictions as a way to reduce body weight and optimize sports performance. Paradoxically, in this study, female athletes consumed more energy than the recommendations.

Carbohydrates are essential for athletes because they contribute to meeting their specific energy needs, to maintain glycemia and recover glycogen reserves [28]. Furthermore, inadequate carbohydrate intake could result in the use of body protein as an energy source, impairing the growth and development processes in both sexes [37]. Additional studies should be carried out to assess whether insufficient intake of energy and carbohydrates, according to ADA recommendations [10], impairs either the growth or physical performance of young modern pentathletes.

Calcium intakes were below the EAR for both male and female study participants, regardless of age. In surveys carried out in Brazil among adolescent non-athletes [38] and athletes [32, 39, 40], low intakes of calcium according to dietary recommendations were common. Santos et al. [38] reported that given the presence of calcium in many metabolic processes, its deficiency may be noted in several ways, such as muscle numbness, musculoskeletal pains, menstrual cramps, and osteoporosis.

In addition to calcium, dietary iron intake also appears to be inadequate among adolescent athletes [38]. However, in the present study, the average amounts of iron ingested by most athletes of both sexes were in accordance with recommendations [23].

The habit of consuming small snacks by physically active individuals could help meet their energy and nutrient needs, according to Burke et al. [41]. The majority of adolescent athletes in this study concentrated their food intake in the three main meals (breakfast, lunch and dinner). Considering the fact that participants reported a short interval between lunch and the start of training sessions, we suggest an evaluation of using snacks as part of the daily nutritional contribution, mainly during periods of training.

According to Jacobson [42], young athletes normally receive guidance from an unreliable source when it comes to use of supplements, such as from trainers, friends, family, magazines, or television. Our data reinforced these findings; most athletes reported following their trainers’ advice and only a few stated that they used supplements in a manner prescribed by a nutritionist. Energy and hydroelectrolytic supplements were the most frequently used among study participants. Vitamin C supplements, multivitamins, and branched-chain amino acids were also mentioned by a smaller number of athletes.

Vigorous and taxing physical activity together with reduced energy availability may cause adverse effects on pubertal development and reproductive function [43]. Therefore, accurate estimation of individual energy needs is needed to establish appropriate dietary guidelines [44]. In the present study, TEE was estimated by predictive equations. However, we believe that errors might exist in the final values obtained; our research group recently suggested that the FAO/WHO/UNU equation [18] tended to overestimate basal metabolic rate measured by indirect calorimetry [44].

The findings of this work can contribute to awareness among young modern pentathletes of the importance of nutrition and the role of each nutrient, for adequate physical performance, muscular recovery, health preservation, and promoting growth and development. Our results will also help sports nutrition professionals in advising adolescent pentathletes.

A main limitation of this study is that we were unable to obtain a homogeneous distribution of athletes at each stage of sexual maturity, so as to more accurately investigate the influence of this variable on eating habits. In addition, the analyses performed here might provide more useful information if conducted using a larger sample size. Further studies will be carried out that are focused on this sport, especially regarding the nutritional demands of athletes during each pentathlon event.

## Conclusions

The adolescent modern pentathlon athletes in this study had inadequate eating habits with respect to energy, carbohydrates, and calcium intake. Moreover, the majority of athletes made use of supplements, even without qualified nutritional counseling, and showed qualitative inadequacy in their eating habits, especially with regard to frequent consumption of soft drinks and low consumption of fruits and vegetables.
